# The Utility of Specific Antibodies Against SARS-CoV-2 in Laboratory Diagnosis

**DOI:** 10.3389/fmicb.2020.603058

**Published:** 2021-01-13

**Authors:** Yu Fu, Yunbao Pan, Zhiqiang Li, Yirong Li

**Affiliations:** ^1^Department of Clinical Laboratory, Zhongnan Hospital, Wuhan University, Wuhan, China; ^2^Department of Neurosurgery, Zhongnan Hospital, Wuhan University, Wuhan, China

**Keywords:** COVID-19, SARS-CoV-2, specific antibodies, dynamic change, serological assays

## Abstract

The Coronavirus Disease 2019 (COVID-19) caused by the severe acute respiratory syndrome coronavirus-2 (SARS-CoV-2) has now become a global pandemic due to its high transmissibility. The unavoidable shortcomings of traditional diagnostic assay, including nucleic acid testing, diverse serological assays characterized by high-throughput and less workload, are playing a more and more crucial role to supplement the nucleic acid test. In this review, we summarize the dynamic change of the specific IgM, IgG, and IgA antibodies against SARS-CoV-2 as well as neutralizing antibodies and discuss the clinical utility and limitations of the different serological assays. SARS-CoV-2, a newly discovered virus, shows some unique pathogenetic and epidemiological characteristics that have not been completely understood so far. Currently, studies about the antibody responses against SARS-CoV-2 and the clinical utility of serological testing are increasing. It’s well suggested that the combination of serological tests and nucleic acid tests can cohesively improve the testing efficiency for identifying COVID-19 suspected patients.

## Introduction

In December 2019, several cases of unknown pneumonia accompanied by respiratory syndromes were reported in Wuhan, Hubei, China (Chen N. et al., 2020). Subsequently, a novel coronavirus was identified in respiratory samples obtained from these patients with unknown pneumonia and the causative agent has currently been named severe acute respiratory syndrome coronavirus 2 (SARS-CoV-2) (Guan et al., 2020). As of November 22, 2020, about 58 million confirmed cases have been reported worldwide with a total number of 1.3 million deaths (available from https://www.worldometers.info/coronavirus/?fbclid=IwAR2WhtIvODVjwxZEszArsVM0ypi0ZJvQ3SVjdnjuyl9ViV2IZPnIdKS5rto). Most COVID-19 patients show mild respiratory symptoms, while some severe cases might present acute respiratory distress syndrome (ARDS), septic shock, and multiple organ failure (Wang et al., 2020). It’s reported that the fatality rate of COVID-19 was approximately 4% (Zhang et al., 2020d), which is obviously lower than that of SARS (9.5%) and much lower than that of MERS (34.4%) (Petrosillo et al., 2020). Severe Acute Respiratory Syndrome (SARS) caused by SARS-CoV occurred during 2002–2003 and developed into a global pandemic with high infection rates among humans (Hu et al., 2017). MERS-CoV was first identified in Saudi Arabia and has now spread to the whole world with a high fatality rate, and it’s still not stopped yet (Bleibtreu et al., 2020).

SARS-CoV-2 is an enveloped, single-stranded RNA virus with oval or round particles that measure about 50–200 nm in diameter (Shereen et al., 2020). The major structural proteins of SARS-CoV-2 are the spike surface glycoprotein (S), a small envelope protein (E), matrix protein (M), and nucleocapsid protein (N), respectively. The M and E proteins play crucial roles in virus assembly (Malik, 2020). The spike protein (S) of coronavirus, a type I transmembrane glycoprotein, was found to have the ability of binding to the host cell surface receptor angiotensin-converting enzyme 2 (ACE2), followed by entering host cells (Wrapp et al., 2020). The N protein that binds to viral RNA is in the central role of the transcription and replication of RNA and can influence the cell cycle processes of host cells. It is widely accepted that IgM provides the first line of defense during microbial infections, before the generation of adaptive, high-affinity IgG responses that are important for long-lived immunity and immunological memory (Racine and Winslow, 2009). Both the S and N proteins are the important antigens of SARS-CoV-2 and many serological diagnostic tests have been developed based on the specific IgM and IgG antibodies against the S and the N proteins (Woo et al., 2005). Many publications have analyzed specific IgM, IgG, and IgA antibodies against the N and the S proteins in COVID-19 patients. Therefore, we summarize the kinetics of antibody response to SARS-CoV-2 in patients with COVID-19 including the specific IgM, IgG, and IgA against the S and the N proteins, to help us evaluate and employ the serological testing assays rapidly, and analyze the testing results reasonably, which help better contain the spread of the causative agent of SARS-CoV-2.

In this review, we first focus on the current knowledge regarding the humoral response to COVID-19 infection including the profiles of IgM, IgG, IgA, and neutralizing antibodies and then summarize the characteristics of antibodies dynamic change during the whole disease progression. We also discuss the diverse serological assays based on different antigens of SARS-CoV-2 and the limitations of the serodiagnosis. We strongly believe that those serological dynamic assays with high quality assurance system are crucial to facilitate better containment of the epidemic.

## The Dynamic Change of Specific Antibody Against SARS-CoV-2 in Patients With COVID-19

### The Seroconversion Time of Specific IgM and IgG Antibodies Against SARS-CoV-2

Previous studies revealed that the median seroconversion time of specific IgM and IgG against SARS-CoV-2 varies differently, ranging from 5 to 13 days, 11 to 14 days, respectively, after symptom onset (Gaddi et al., 2020; Guo L. et al., 2020; Long et al., 2020a; Sun et al., 2020; Xiang F. et al., 2020; Zhao et al., 2020). There exist three types of seroconversion including synchronous seroconversion of IgG and IgM (34.6%, 9/26), IgM seroconversion earlier than that of IgG (26.9%, 7/26), and IgM seroconversion later than that of IgG (38.5%, 10/26) according to a longitudinal observation (Long et al., 2020a). In our previous serological tests, the seroconversion of both IgM and IgG occurred at day 4 by colloidal gold-based immunochromatographic (ICG) strip among the confirmed cohort (Pan et al., 2020), which was 1 day earlier than that reported by Zhang et al. (2020c) using ELISA assay. The seroconversion of specific IgM and IgG varies against different types of antigens from SARS-CoV-2. More patients had earlier seroconversion for IgG than IgM against N proteins and RBD; the seroconversion of IgM in severe COVID-19 cases was delayed compared to IgG (Shen L. et al., 2020; To et al., 2020b). Thus, the delayed development of specific IgM antibodies could be a sign for severe patients with COVID-19.

By contrast, in SARS-CoV infections, specific IgM, IgG, or IgA antibodies against SARS-CoV were all tested negative until at least 3 days after symptoms onset and all positive after at least 19 days (Hsueh et al., 2004). In MERS infection, antibody response to MERS is commonly detected in weeks 2–3 after onset (Alshukairi et al., 2016; Corman et al., 2016).

The seroconversion of specific IgM and IgG antibodies against SARS-CoV-2 mostly turn positive in the second or third week after symptom onset and vary greatly when involved with different specific types of antigens. Consequently, we should pay more attention to the seroconversion time of specific IgM and IgG against SARS-CoV-2 in each different individual with COVID-19 to improve the accuracy and precision of serological assays.

### The Dynamic Change of Specific IgM and IgG Antibodies in SARS-CoV-2 Infections Over the Whole Disease Progression

**FIGURE 1:**
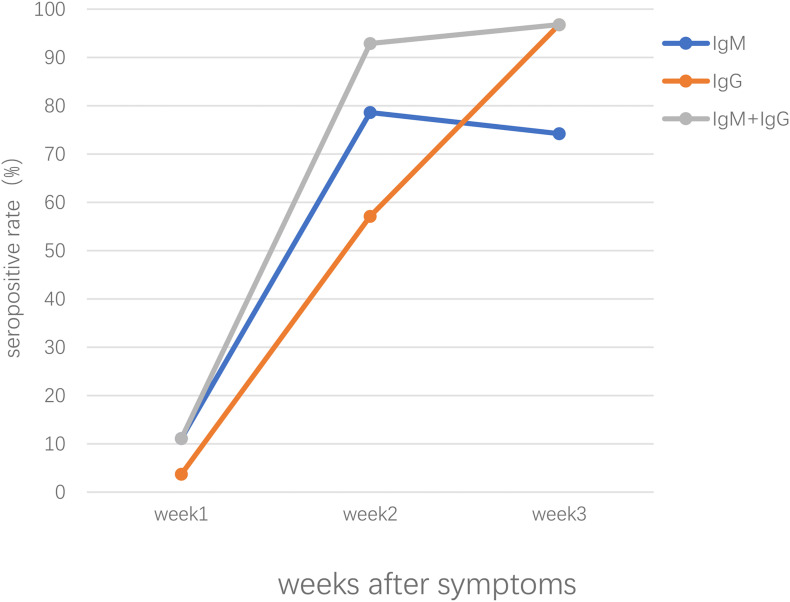
The seropositive rates of specific IgM, IgG, the combination of IgM and IgG antibody response in patients confirmed with SARS-CoV-2 infection after symptoms onset.

Our previous study conducted in Zhongnan Hospital of Wuhan University, China, showed that both IgM and IgG were first detected at day 4 and the positive rate of IgM and IgG were 11.1% and 3.6%, respectively, in patients of early stages; in intermediate and late stages, the positive rate remained about 75% and increased up to 96.8% for IgM and IgG, respectively (Pan et al., 2020). Patients confirmed with COVID-19 showed positive results of virus-specific IgM reaching a peak around 18–22 days post COVID-19 onset, specific IgG around 17–19 days (Long et al., 2020a; Xiang F. et al., 2020; Zhao et al., 2020). As shown in Figure 1, the seropositive percentages of specific IgM, IgG, and the combination of IgM and IgG in patients confirmed with COVID-19 varied over time (Pan et al., 2020). A study enrolled with 105 COVID-19 patients and 197 non-COVID-19 patients demonstrated that IgM reached peak within 15–21 days and slowly began to decline, while IgG peaked during 22–39 days and lasted for a longer time (Wu L. X. et al., 2020). Another study focused on the early humoral response to SARS-CoV-2 showed that IgM antibody level rose between days 8 and 14 but didn’t increase further between days 15 and 21 or after day 21; however, the IgG antibodies were detected on days 0–7, increased on days 8–14 and kept on rising until days 15–21, reaching a plateau by day 21 (Guo L. et al., 2020). Another study demonstrated that the level of IgM rose above the baseline level at day 6 (seroconversion time), peaked at around day 18, and fell to below the baseline level at about day 36, while IgG was seropositive by day 3, peaked at around 23 days, and then maintained at relatively high levels (Shu et al., 2020).

Among most non-ICU patients, total antibodies increased sharply since the first week and elevated during the next 2 weeks; specifically, N-IgM shared a similar dynamic pattern with N-IgG in the first 2 weeks in the same patient, IgM continued to increase until the third week and yet, the level of IgG exceeded IgM in the 2–3 week after onset, indicating that there was an IgM to IgG class switch (Sun et al., 2020; Zhao et al., 2020). Nevertheless, it’s the level of S-IgG that was much higher in COVID-19 patients with mild symptoms compared to another antigen and therefore it’s speculated that S-IgG plays a more important role in clearing the viruses and protecting patients (Sun et al., 2020). IgG antibodies generally keep positive for a long period time, whereas 3.2% (3/95) cases confirmed with COVID-19 showed IgG antibodies turning from positive to negative in just 5 days (Shu et al., 2020). It’s reported that 53.1% of mild cases were tested IgM negative, while only 14.3% negative in severe ones (Liu et al., 2020). Patients with severe COVID-19 tend to produce more robust humoral antibody response than those with mild ones. Other groups also reported that severe cases generally had an earlier SARS-CoV-2 specific IgM response and higher peak measurements compared with mild cases (Long et al., 2020a; Lynch et al., 2020; Perera et al., 2020; Zhao et al., 2020). Thus, the high titer of total Ab is probably the potential risk factor of worse clinical prognosis of COVID-19 patients. Notably, there have been reported that the seroconversion of mild COVID-19 cases took at least 4 weeks, which was longer than severe cases, and a small number of COVID-19 patients remained seronegative for Ab testing during the whole hospitalization period (Zhao et al., 2020). Guo X. et al. (2020) reported a case of mild COVID-19 with long virus-carrying time, which may be related to the weak production of virus-specific IgG and IgM, and recurrence of positive SARS-CoV-2 RNA occurred in stool specimens after discharge possibly due to intermittent virus shedding. Therefore, it’s necessary to apply multiple tests in order to make final correct diagnosis instead of just ignoring probable patients tested specific antibodies negative. What’s more, the serological assays tested positive for IgG and IgM were 81.1% (30/37) and 62.2% (23/37), respectively, in asymptomatic patients with COVID-19, which were obviously lower than 83.8% (31/37) for IgG and 78.4% (29/37) for IgM in the symptomatic COVID-19 patients (Long et al., 2020b).

The profiles of virus-specific antibodies vary in different individuals and further studies are needed to explore the uncertainties about the characteristics of immunological response to SARS-CoV-2.

By contrast, in SARS associated infections, nearly all SARS patients show virus-specific IgM and IgG antibody by week 3, which began to decrease in the 3–4 week after illness onset, and IgM remained at a low level in a few weeks, while the concentrations of IgG antibody that is 4∼8 times higher than that of IgM antibody, reaching its peak at 20 days after infection, and then maintained in a high level for a long time (Woo et al., 2004; Mo et al., 2005). There had been reported that an early MERS antibody response is correlated to the reduction of infection severity and the concentration of IgG antibody of most patients can reach a peak in the third week (Park et al., 2015).

### The Development of the Secretory IgA Response May Play a Crucial Role in Preventing SARS-CoV-2 Infections

Current serological tests mainly revolve around the detections of specific IgM, IgG, or total immunoglobins. However, IgA plays a crucial part in mucosal immunity and is considered as the most powerful immunoglobin to fight infectious pathogens in respiratory and digestive systems, which can neutralize SARS-CoV-2 throughout virus entry (Chao et al., 2020). In a study, IgA seroconversion occurred in the first week in 3/4 (75%) patients after the disease onset, and the levels of IgA were persistently higher than IgM antibodies during the whole observation period, with a peak level at 20–22 days, which was later than that of IgM (Padoan et al., 2020b). Another study showed that the first seroconversion day of IgA was 2 days after onset of the initial symptoms and the levels of specific IgA noticeably increased about ∼2 weeks after the symptom onset and remained elevated for the following 14 days after seroconversion, which were significantly higher than those of IgM in both severe and non-severe patients (Yu et al., 2020). Guo L. et al. (2020) reported that both IgA and IgM increased between days 8 and 14 but did not rise further between days 15 and 21 or after day 21. It is similar to the observation in another study done about SARS-CoV associated infections that IgM and IgA shared similar dynamic patterns including seroconversion and antibody titers (Hsueh et al., 2004).

### The Development of Neutralizing Antibodies (nAbs) During Infections by SARS-CoV-2

It’s reported that RBD-specific antibodies have greater potency to neutralize infection and thus the RBD of SARS-CoV-2 can serve as an important target for the development of potent and specific nAbs (Jiang et al., 2020). Wu F. et al. (2020) found that NAbs were detected in patients from days 4 to 6 and reached peak levels from days 10 to 15 after disease onset. What’s more, a SARS-CoV RBD-specific human neutralizing nAb, CR3022, could bind SARS-CoV-2 RBD with high affinity and recognize an epitope on the RBD that does not overlap with the ACE2-binding site while the neutralization capacity remains unclear (Tian et al., 2020). Whereas, findings in another study noted that high levels of nAbs may be the consequence of strong inflammation or innate immune response in older patients who developed higher nAbs titers and tend to have worse outcomes after SARS-CoV-2 infection (Wu F. et al., 2020). Therefore, the neutralizing antibodies maybe don’t necessarily play the protective roles in the illness as supposed. However, one pilot study on convalescent plasma with a high concentration of neutralizing antibodies can rapidly reduce the viral load and tends to improve clinical outcomes of patients infected with SARS-CoV-2 (Duan et al., 2020). To summarize, the therapeutic efficacy and effects of neutralizing antibodies need to be elucidated with further proofs and clinical evidence.

## The Serological Tests for Specific IgM and IgG Antibodies

Many serological tests based on different antigens of SARS-CoV-2 have been developed in order to confirm suspected patients and to exclude patients infected with other respiratory viruses, thereby facilitating the control of this global pandemic. Some of the most commonly used immunoassays were listed in Table 1.

**TABLE 1:** The sensitivity and specificity of IgM and IgG detection in different serological tests.

	Sensitivity (%)		Specificity (%)		Antigen used	References	Advantages	Disadvantages
	IgM	IgG	IgM	IgG				
GICA	88.66*		90.63*		Recombinant antigen (MK201027)	Li et al., 2020	Rapid, flexible and accurate testing, low cost, and being less time-consuming	False positive, qualitative not quantitative
	71.1*		96.2*		Synthetic antigens of the S, M, and N proteins	Shen B. et al., 2020		
	57.1	81.3	100	100	Recombinant antigen of new coronavirus	Zhang et al., 2020a		
	100*		93.3*		SARS-CoV-2 NP	Huang C. et al., 2020		
ELISA	44.4	82.54	100	100	Recombinant antigen of new coronavirus	Zhang et al., 2020a	High-throughput, less turn-around time, small sample consumption	Endogenous interference, poor repeatability
	77.3	83.3	100	95	The recombinant N protein of SARS-CoV-2	Xiang F. et al., 2020		
	70.8	92.5	*NA*	*NA*	SARSr-CoV Rp3 nucleocapsid protein (NP)	Shu et al., 2020		
FICA	98.68	98.72	93.1	100	The recombinant nucleocapsid protein	Feng et al., 2020	High sensitivity and specificity, accurate quantitative detection	Higher requirements for instruments
	75.6*		100*		SARS-CoV-2 nucleocapsid protein (NP)	Xiang J. et al., 2020		
	87.28	90.17	94	96.72	N and S1 protein	Diao et al., 2020		
CLIA	78.65	91.21	97.5	97.3	NA	Padoan et al., 2020a	Easy operation, high sensitivity, large population screening	Poor selectivity, strict external factors needed
	48.1	88.9	100	90.9	N and S protein	Jin et al., 2020		
	96.8	96.8	92.3	99.8	Highly purified RBD of the S protein	Ma et al., 2020		
	80	90	95	95	The combined N and S glycoproteins	Qu et al., 2020		

ELISA, enzyme-Linked immunosorbent assay; GICA, colloidal gold immunochromatographic assay; FICA, fluorescence immunochromatographic assay; CLIA, Chemiluminescence Immunoassay. NA, not available. *Means the sensitivity and specificity for the combination of IgM and IgG.

### Colloidal Gold Immunochromatographic Assay

Colloidal gold immunochromatographic assay (GICA) is point-of-care testing (POCT) applied for qualitative analysis of target antigen/antibody, which is a feasible method for the diagnosis of COVID-19 in primary hospitals and laboratories, especially in emergency situations with its advantages of straightforward operation, time-saving and clinically compatible steps, small sample consumption, and easy result interpretation (Kim et al., 2015; Li et al., 2019; Huang C. et al., 2020). The N-S recombinant protein-based GICA assay for testing SARS-CoV-2 specific IgM and IgG antibodies demonstrates the strip possesses high specificity and is highly reproducible in sample results (Shen L. et al., 2020). IgG/IgM Rapid Test, manufactured by Cellex Inc., on April 1, 2020, is an aid in the diagnosis of patients with suspected SARS-CoV-2 infections (FDA, 2020). Our previous research found that the positive rates of IgM and IgG were relatively low in the early stage (1–7 days from onset) of SARS-CoV-2 infection, and gradually increased in the intermediate stage (8–14 days from onset) and peaked at a late stage (more than 15 days), and the combination of IgM and IgG results dramatically increased the sensitivity of GICA tests (Pan et al., 2020). Other studies reported the sensitivity and specificity of the colloidal gold immunochromatography assay for SARS-CoV-2 specific IgM/IgG antibody using synthetic antigens of the S, M, and N proteins were 71.1 and 96.2%, respectively (Shen B. et al., 2020). Huang S. et al. (2020) found that the sensitivity of the combined GICA IgM and IgG detection was 75/91 (82.4%) and were negative for healthy controls with a specificity of 100%. Zhang et al. (2020b) evaluated and found that the eukaryotic expression spike proteins are more suitable than the prokaryotic expression nucleocapsid proteins for the proposed GICA.

In conclusion, GICA is a good diagnostic tool for large population screening and community surveillance for identification of SARS-CoV-2. It’s supplementary to the nucleic acid detection by RT-PCR and provides qualitative results compared to ELISA assay showing quantitative antibody titer (Gaddi et al., 2020).

### Enzyme-Linked Immunosorbent Assay

Enzyme-linked immunosorbent assay (ELISA) is a common biomedical assay that utilizes enzyme-labeled antigens/antibodies to detect specific molecules in specimens and is featured by cost-saving, easy operation (Gan and Patel, 2013). IgM and IgG antibodies against SARS-CoV-2 could be detected in the middle and later stages of the disease by ELISA-based serology tests, and seroconversion of specific IgM and IgG antibodies was observed as early as the fourth day after the onset of symptoms by ELISA (Long et al., 2020a). In addition, among the patients confirmed with COVID-19, the sensitivity, specificity, PPV, NPV, and consistency rate of IgM were 77.3% (51/66), 100%, 100%, 80.0%, and 88.1%, respectively, and IgG was 83.3% (55/66), 95.0%, 94.8%, 83.8%, and 88.9%, respectively, using the recombinant N protein of SARS-CoV-2 (Xiang F. et al., 2020). Huang S. et al. (2020) reported the sensitivity of the combined ELISA IgM and IgG detection was 55/63 (87.3%) and had a specificity of 100%. Guo L. et al. (2020) revealed that the detection efficiency of IgM by ELISA was higher than that of qPCR after 5.5 days of symptom onset. Therefore, the combination of IgM ELISA assay with PCR can improve the detective efficacy to identify SARS-CoV-2 infection.

Recently, a microfluidic ELISA system to detect COVID-19 antibodies on a lab-on-chip platform was described and proposed by a research group. Furthermore, this device first separates plasma from whole blood using a microfluidic device and subsequently performs the detection of antibodies in the separated plasma using a semi-automated on-chip ELISA with high-quality plasma and minimal cell interference (Tripathi and Agrawal, 2020). Zhang et al. (2020c) detected specific IgG and IgM from human serum of COVID-19 patients by ELISA using the SARS-CoV-2 Rp3 nucleocapsid protein, which has 90% amino acid sequence homology to other SARS-related viruses. A diagnostic specificity of 100.0% and sensitivity of 98.31% was achieved in a newly established ELISA using 59 sera of infected or vaccinated animals including ferrets, raccoon dogs, hamsters, rabbits, chickens, cattle, and a cat and a total of 220 antibody-negative sera of the same animal species (Wernike et al., 2020). What’s more, Schöler et al. (2020) established a SARS-CoV-2 neutralization assay employing an in-cell ELISA (icELISA) approach. It allows rapid (<48 h in total, read-out in seconds) and automated quantification of COVID-19 infection in cell culture to evaluate the efficacy of NAbs and antiviral drugs using reagents and equipment present in most routine diagnostics departments (Schöler et al., 2020). A report on a microfluidic, multiplexed POC test based on multiple SARS-CoV-2 antigens—S, N, and RBD—shows good concordance with a live virus microneutralization assay and successfully tracked the longitudinal evolution of the antibody response in infected individuals (Heggestad et al., 2020).

### Fluorescence Immunochromatographic Assay

Fluorescence immunochromatographic assay (FICA) designed for the detection of specific immunoglobulins in blood/serum is based on the combination of immunofluorescence technique and chromatography technology, showing high sensitivity, accurate quantitative detection, and good stability (Sotnikov et al., 2017).

It’s reported that the sensitivity and specificity of the FICA with Lanthanide, Eu (III) using the recombinant nucleocapsid protein as antigen were 98.72% and 100% (IgG), and 98.68% and 93.1% (IgM), respectively (Feng et al., 2020). In other studies, it’s concluded that fluorescence immunochromatography is much easier for quantitative detection and more sensitive and specific in serodiagnosis than the traditional colloidal gold immunochromatographic assay (Xie et al., 2014; Chen Z. et al., 2020).

### Chemiluminescence Immunoassay

Chemiluminescence immunoassay (CLIA) is based on double-antibodies sandwich immunoassay to detect specific antibodies through amplified signals of chemical luminescence materials. With the features of easy operation and high sensitivity, it is in the central role of the early diagnosis of diseases and large population screening (Min et al., 2018). Padoan et al. (2020a) found that the sensitivities and specificities of CLIA were 78.65%, 97.5% (IgM), and 91.21%, 97.3% (IgG). Another study using N and S proteins calculated that the sensitivities and specificities of serum IgM and IgG antibodies were 48.1% and 100%, 88.9% and 90.9%, respectively (Jin et al., 2020). A systematic review and meta-analysis summarized that the pooled sensitivity and specificity of CLIA were 97.8% and 98%, respectively, and the sensitivity increased 3 weeks after symptom onset (Lisboa Bastos et al., 2020). Cai et al. (2020) developed a peptide-based chemiluminescent immunoassay using synthetic peptide antigens including the orf1a/b, S, and N proteins to detect the positive rate of immunoglobulin G and IgM, which were 71.4% and 57.2%, respectively, among patients infected with SARS-CoV-2. CLIA detecting antibodies against RBD is considered the best diagnostic test accuracy (Mekonnen et al., 2020). Thus, it is a reliable immunoassay for assessing the immunological response in patients infected with SARS-CoV-2 and the detective accuracy of SARS-CoV-2 can be enhanced by the combination of nucleotide assay RT-PCR.

## Limitations About Serological Testing for COVID-19 Patients

### The Duration of the Window Period of Specific IgM and IgG Antibodies Alter in Different Patients

Antibody responses to infection take days to weeks to be reliably detectable and the levels of those antibodies decrease over time. It’s reasonable that in early phases of SARS-CoV-2 infections, the levels of specific IgM and IgG antibodies are too low to be detected in the serum or plasma samples leading to false-negative results of serological tests. There is concern that virus-specific antibodies detection may miss cases due to a larger window of time for indirectly detecting SARS-CoV-2 (Udugama et al., 2020). More than 60% of infected individuals are seronegative (IgM or IgG) in the first 7 days (Zhao et al., 2020). IgM and IgG converted to negative around 36 days and over 50 days separately (Guo L. et al., 2020). Notably, there have been reports that the seroconversion of mild COVID-19 cases took at least 4 weeks, which was longer than severe cases, and a small number of COVID-19 patients remained seronegative for Ab testing during the whole hospitalization period (Zhao et al., 2020), and it’s assumed that the innate immune response of these mild patients cleared the virus before the humoral immune system produced any antibodies. Serology measures the host response to infection and is an indirect measure of infection that is best utilized retrospectively (Tang et al., 2020).

The combination of serological tests and nucleotide acid assays is essential to improve the sensitivity and specificity of clinical diagnosis for COVID-19.

### The Cross-Reactivity Between SARS-CoV-2 and Other Endemic Human Coronaviruses

It’s widely accepted that cross-reactivity has a great impact on the sensitivity and specificity of the serological tests. A recent study has demonstrated negligible cross-reactivity from human 257 coronavirus, NL63, to SARS-CoV-2 (Amanat and Krammer, 2020). It’s reported that sera from the healthy donors were found to have SARS-CoV-2 neutralization activity comparable to that of samples from seropositive patients with COVID-19 (van der Heide, 2020). Lv et al. (2020) established models of mice infected with SARS-CoV-2 and their results showed that cross-reactivity in antibody binding to the spike protein is common, and cross-neutralization of the live viruses may be singular, revealing non-neutralizing antibody response to conserved epitopes in the spike protein. Long et al. (2020a) did observe in a study enrolled with a total of 285 patients with COVID-19 showed cross-reactivity to the nucleocapsid proteins of SARS-CoV-2. Therefore, other endemic human coronaviruses may still have impact on accurately diagnosing patients with real SARS-CoV-2 infections (Lee et al., 2020).

### There Exist Substances Interfering With the Serological Tests Results

Endogenous components in the fingerstick blood or the plasma/serum of venous blood sample can have an effect on the immunoassays of specific antibodies. Notably, endogenous heterophilic antibodies are present in many serum samples to interfere with two-site ELISAs, thereby evoking false-positive signals as well as the human autoantibodies rheumatoid factors (Grebenchtchikov et al., 2002; Berth and Willaert, 2016). Hemolysis, the breakdown of erythrocytes with subsequent release of the intracellular contents, can frequently interfere with the serology tests, leading to either positive or a negative bias in test results (Snyder et al., 2004). For instance, three children diagnosed with Kawasaki disease in Hong Kong, China, with no communication contacts with COVID-19 patients, were tested positive against anti-RBD and anti-NP antibodies of SARS-CoV-2, yet negative for SARS-CoV-2 NPA PCR and neutralizing antibodies, indicating a false-positive antibodies result (To et al., 2020a). Another study found that the rate of positive antibodies against N-protein and S-RBD was 3.6 and 1.6%, respectively, among healthy and non-COVID-19 individuals, revealing that the positivity of N-protein and S-RBD don’t necessarily determine SARS-CoV-2 infection (McAndrews et al., 2020).

## Discussion

The outbreak of Coronavirus disease 2019 (COVID-19) caused by SARS-CoV-2 made urgent and necessary the need for diagnostic tests that can identify COVID-19 patients. The dynamic profiles of specific IgM and IgG antibody against SARS-CoV-2 in patients with COVID-19 varies differently. The median time for seroconversion of specific IgM and IgG antibodies is about 9–14 days; however, early seroconversion has been reported at 3–5 days (Gaddi et al., 2020; Long et al., 2020a; Sun et al., 2020; Xiang F. et al., 2020; Zhao et al., 2020). It’s found that the IgM level lasted more than 1 month, indicating a prolonged stage of virus replication in SARS-CoV-2-infected patients, and the IgG levels generally increased only in the later stages of the disease (Dhama et al., 2020).

Apart from specific IgM and IgG antibodies, the secretory IgA is considered to be powerful in mucosal immunity to eliminate the invasive virus SARS-CoV-2 and the seroconversion of IgA occurred commonly earlier than other antibodies. High levels of neutralizing antibodies are assumed to be associated with the overreaction of innate immune response or strong inflammation and don’t necessarily play the protective role in SARS-CoV-2 infection. The therapeutic efficacy and effects of neutralizing antibodies need to be furthermore elucidated. The dynamic changes in the antibody level of COVID-19 patients can provide more clinical information about the illness progression so that medical staff can cope with COVID-19 patients in a more proper and efficient way. Testing with IgG positive indicates prior infections with the virus and does not necessarily mean protective immunity in infected individuals; what’s more, it’s very likely to be IgG positive while shedding virus as determined by molecular assays due to the timing of infection stages and serum/plasma sampling (Theel et al., 2020). In conclusion, at least three situations could happen in serological test results: (1) seropositive but negative for molecular genetic assay results reflecting clearance of an earlier, milder infection, (2) positive result from molecular genetic assays for SARS-CoV-2 infection are seronegative due to the lag in antibody production following infection, and (3) limitation in sensitivity and specificity of serological tests including false positive and negative results, which may have undesirable impact on the socioeconomic decisions and overall public confidence in the results (Carter et al., 2020). Strict quarantine and health surveillance should be taken for all COVID-19 patients, even discharged to prevent a potential virus spread. It is still blurred whether patients with COVID-19 infection would acquire permanent immunity to this disease after certain time, and further studies need to be done about this. In the aspect of detective assays, one of the drawbacks of serological tests is the limited sensitivity in the window period at early stages; choosing different target antigens also has impact on sensitivity and specificity of serological assays (Caruana et al., 2020). A vast number of serological tests have been developed on the market for the detection of SARS-CoV-2 infection. Lateral flow assay (LFAs) such as GICA and FICA often demonstrates lower sensitivity than ELISAs and CLIAs (Koczula and Gallotta, 2016). LFAs are powerful diagnostic tools for large scale population screening and community surveillance for identification of SARS-CoV-2. Numerous LFIA based rapid POC tests have been developed by several companies, which enable the detection of IgM and IgG antibodies produced in suspects in response to SARS-CoV-2 infection (Vashist, 2020). It does not require any instrument or trained staff and, thus, it can be employed at any place and time, especially in developing nations with limited healthcare resources and remote settings. The assay is ideal for primary healthcare workers for the rapid testing of COVID-19 suspects (Vashist, 2020). LFIA method could be helpful in assessing in short time the possible contagiousness of subjects who, due to work needs, cannot guarantee “social distancing” to avoid the spread of COVID-19 by symptomatic and above all by asymptomatic individuals (Noce et al., 2020). Both ELISAs and CLIAs are reliable to assess the immune response of patients infected with SARS-CoV-2 (Gaddi et al., 2020; Guo L. et al., 2020; Jin et al., 2020). In specific clinical situations, due to the limitations about serological testing, choosing proper serological tests can greatly improve the testing efficiency. In general, antibody detection for SARS-CoV-2 with the advantages of high-throughput, faster turn-around time, and less work labor can effectively compensate for the shortcomings of nucleic acid detection, and assist nucleic acid testing to confirm the diagnosis of suspected patients. Therefore, the combination of nucleic acid test and serological tests based on different antigen proteins play a crucial role to improve the diagnostic accuracy and better contain the spread of SARS-CoV-2.

In conclusion, antibody testing can be used as a supplement to the SARS-CoV-2 nucleic acid testing method to confirm and exclude COVID-19. The exact role of the antibody against SARS-CoV-2 in the clinical diagnosis, treatment, and prognosis judgment of COVID-19 needs further study on the pathogenic mechanism of SARS-CoV-2.

## Author Contributions

YF performed the data analyses and wrote the manuscript. YP contributed to the conception of the study. ZL and YL helped revise the draft and were responsible for the accuracy of this manuscript. All authors contributed to the article and approved the submitted version.

## Conflict of Interest

The authors declare that the research was conducted in the absence of any commercial or financial relationships that could be construed as a potential conflict of interest.
